# A case of blepharitis caused by *Trichophyton rubrum*^☆^

**DOI:** 10.1016/j.abd.2020.08.037

**Published:** 2022-02-24

**Authors:** Congcong Zhang, Hao Chen

**Affiliations:** Institute of Dermatology, Chinese Academy of Medical Sciences and Peking Union Medical College, Nanging, China

Dear Editor,

A 10-year-old boy was referred to our clinic with symptoms consisting of two-week-old itchy erythema on periocular skin and multiple 3-day-old papulopustules on the left upper palpebra. The patient had animal contact about one month ago and the lesions were not treated previously. Physical examination revealed erythema with mild scales on the periocular skin and several sesame-sized milky papulopustules on his left upper palpebra (Fig. 1). Routine laboratory findings including blood and urine, hepatorenal function and immunity items were normal. Because the subject had contact with his dog, a mycological examination was performed. Multiple fungal hyphae and white fuzzy-like colonies were found. These were grown in culture and were identified as the *Trichophyton rubrum* (Fig. 2). Microscopically, clublike macroconidia and microconidia spores were also found (Fig. 3). The final diagnosis of blepharitis caused by *Trichophyton rubrum* was confirmed. The patient received treatment with oral itraconazole 100 mg per day for two weeks and topical bifonazole cream once every night for four weeks. Full recovery was observed and no recurrence during the two-month follow-ups was noted.

**Figure 1:**
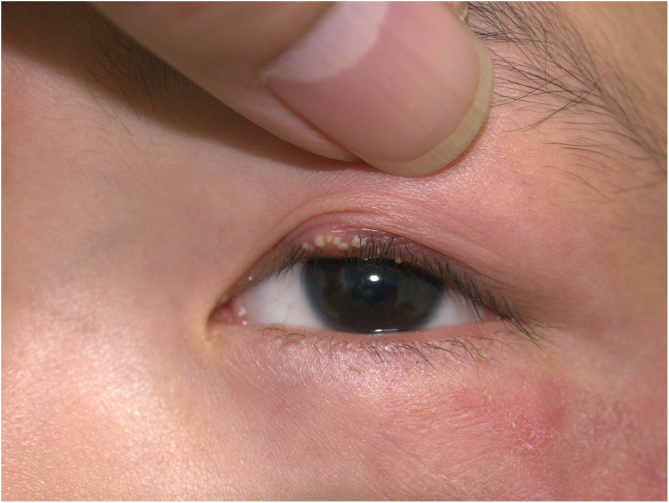
Clinical presentation: erythema with mildly scales on the periocular skin and sesame-sized milky papulopustules on the left upper palpebra.

**Figure 2:**
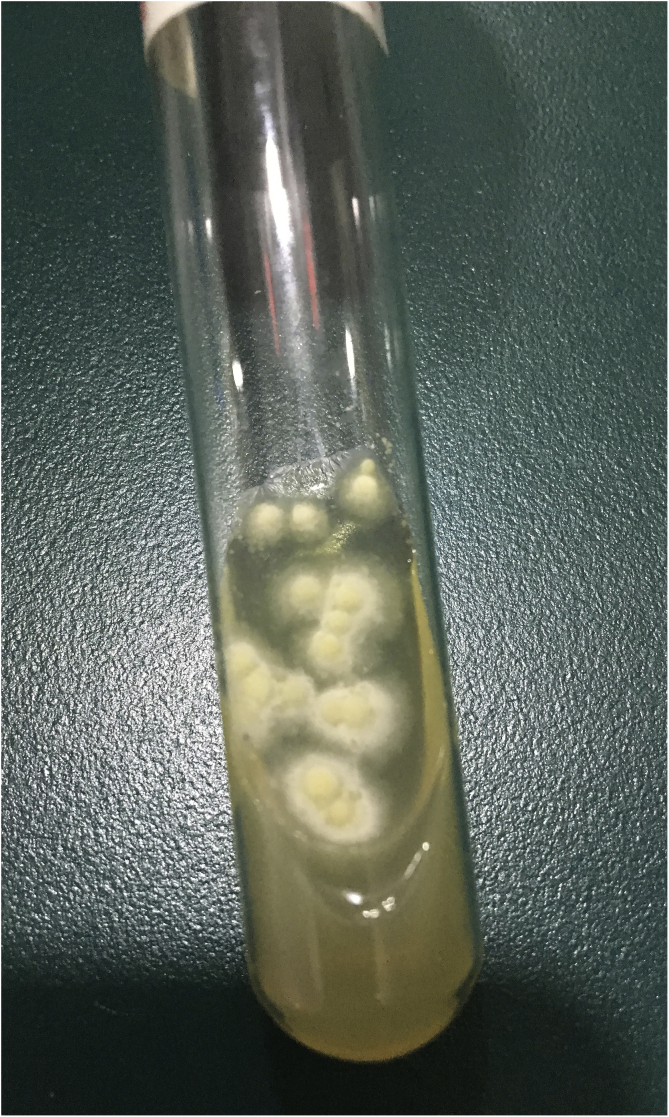
The colonies of fungal culture.

**Figure 3:**
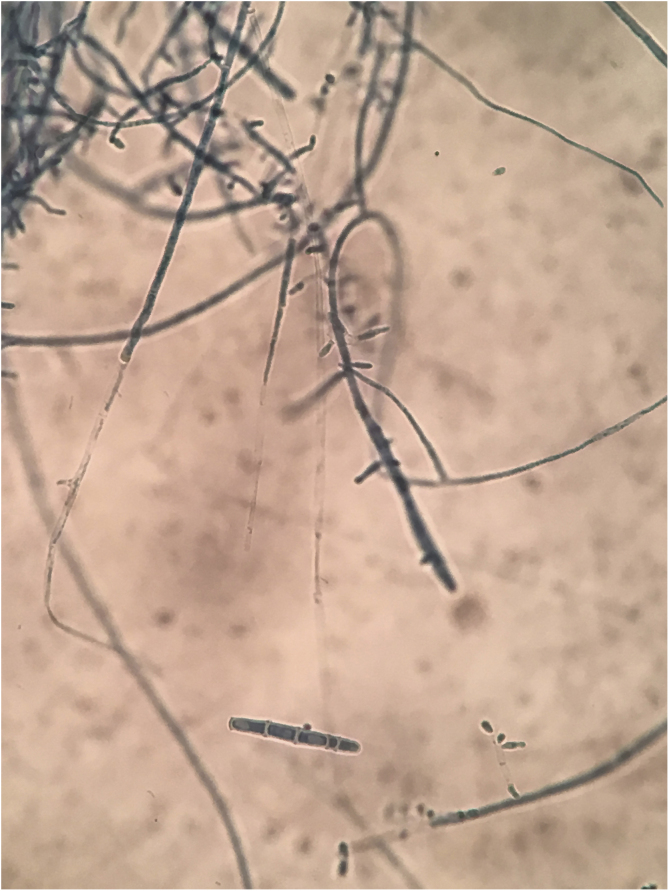
Microscopic examination showed clublike macroconidia and microconidia spores.

Dermatophytosis is a common superficial fungal infection and is usually caused by *Trichophyton rubrum*. Feet, trunk, and nails are the most affected sites. The features of a typical lesion consist of central clearing surrounded by an advancing, red, scaly, elevated border, and vesicles can also appear on the border of the affected area with increased inflammation.^1^ Blepharitis is a chronic inflammatory disorder with complex symptoms of the eyelids and is usually caused by bacterial colonization and *Demodex*.^2^ However, some of the reported studies suggest that it could be of fungal origin as well.^3^ Noticeably, the sesame-sized milky papulopustules of our case were different from these typical features. As the symptoms were identified in the early stages, the papulopustules and periocular scaling responded well to the short-time treatments. *Trichophyton* can induce different clinical features mimicking other conditions, such as impetigo, eczematous dermatitis, and lupus erythematosus.^4^ The site of the invasion, variable invasive capacity, individual immunity, diagnosis, and treatment procedures may associate it with various clinical manifestations and can easily lead to misdiagnosis and wrong treatment. False treatments like topical steroids could further cause an eruption of the lesions and more serious consequences. Therefore, early identification of medical history and precise diagnosis are the key aspects to prevent delayed healing or chronicity. Once identified, systemic and adequate duration of the treatment with oral antifungal agents are required. To prevent relapses, domestic animals and dermatophytosis from other body parts should be examined and treated. The present report reinforces the rare clinical features of fungal blepharitis and the importance of medical history in the diagnosis.

## Financial support

CAMS Innovation Fund for Medical Sciences (CIFMS-2017-I2M-1-017), Peking Union Medical College Youth Fund (3332017168) and Six Major Talent Summit in Jiangsu Province (No.WSN-030).

## Authors’ contributions

Congcong Zhang: Article writing; Case analyzing; Patient follow-up.

Hao Chen: History collecting; Picture taking.

## Conflicts of interest

None declared.
